# Medical Implications of Restricting Abortions on Women Diagnosed With Fetal Anomalies Following the Overturn of Roe v. Wade: A Scoping Review

**DOI:** 10.7759/cureus.58994

**Published:** 2024-04-25

**Authors:** Madison Mellquist, Megan Hoedt, Kellie N Fusco, Rachel Alef, Kaitlyn Dittmer, Henry Ash, Wamika Shoukat, Lorenzo Fonteyn, Salome Herzstein, Allie Heineman, Harvey N Mayrovitz

**Affiliations:** 1 Osteopathic Medicine, Nova Southeastern University Dr. Kiran C. Patel College of Osteopathic Medicine, Davie, USA; 2 Osteopathic Medicine, Nova Southeastern University Dr. Kiran C. Patel College of Osteopathic Medicine, Clearwater, USA; 3 Medical Education, Nova Southeastern University Dr. Kiran C. Patel College of Allopathic Medicine, Davie, USA

**Keywords:** roe v. wade, health policy, reproductive rights, abortion ban, fetal anomalies, congenital anomalies, abortion

## Abstract

This scoping review addresses the potential maternal health outcomes of abortion restrictions in the U.S. by studying and analyzing the reported effects of abortion bans or limitations globally. The goal was to examine the medical implications for pregnant women who are unable to abort fetuses that have severe medical anomalies due to imposed restrictions. EMBASE, Medline, and CINAHL databases were searched for studies published in English concerning the medical implications of abortion restrictions in any country prior to the overturn of *Roe v. Wade* in 2022. For the search criteria using Boolean operators, keywords included the terms "fetal anomaly," "abortion ban," and "implications." Inclusion criteria incorporated studies published between 1980 and 2021, and controlled experimental research studies aimed to evaluate interventions were excluded. This resulted in 469 records initially found. Duplicate records were removed, and two separate tier reviews were conducted. Eleven reviewers independently screened abstracts and titles of 332 records to ascertain eligibility. Eligibility included pregnant women diagnosed with fetal anomalies, women denied access to safe abortions, and the maternal and fetal medical impacts of this. Three reviewers in the second screening independently read 36 full articles to further assess eligibility, resulting in 14 articles in the final review. Findings from this study showed that abortion bans in countries around the world have led to health complications in women seeking illegal abortion services, a decline in maternal mental health, including stress and depression, various medical complications such as obstructed labor, and an increase in high-risk fetuses born with severe deficits. The findings of this review portend similar negative consequences to be experienced by women who are subject to stricter abortion laws in the U.S.

## Introduction and background

In June 2022, the U.S. Supreme Court overturned Roe v. Wade, the 1973 court case that was paramount in solidifying the constitutional right to abort a pregnancy before fetal viability [1]. Planned Parenthood v. Casey had previously reaffirmed the precedent set by Roe and further added that the states could not impose an “undue burden” on a woman’s right to choose an abortion pre-viability of the fetus, which is approximately 23-24 weeks of gestation at the earliest [2]. Today, Dobbs v. Jackson Women’s Health Organization [1], the case that overturned Roe v. Wade, has transferred the responsibility of abortion policies and reproductive rights from the federal government back to individual states. Currently, 43 states in the U.S. have placed restrictions on abortions after a certain pregnancy threshold [3]. A scan of fetal anatomy is typically performed around 18-22 weeks gestation to check for anomalies [4]. The results of this scan may indicate the need for “prenatal intervention, postnatal treatment, neonatal palliative care, or elective pregnancy termination” [4]. However, abortion restrictions being implemented prior to the gestational age indicated for proper anatomy scans could limit the possibility of some interventions, which may result in more drastic medical complications for the pregnant woman.

For many women, pregnancy and childbirth are healthy experiences. However, six out of 1,000 pregnancies result in stillbirth, and three out of 1,000 pregnancies are diagnosed with fetal anomalies (also known as birth defects) [5]. Roe protected patients throughout their entire pregnancy by allowing abortion care in cases with complications, such as hemorrhaging, ectopic pregnancies, and pregnancies with fetal anomalies [6]. Many abortion bans are dependent on the gestational age of the fetus [3]. Considering that pregnant women may be unaware of the genetic and anatomical status of the fetus prior to reaching their state’s restriction point, abortion bans may result in them carrying a nonviable pregnancy to term if the pregnancy is not terminated spontaneously, potentially leading to additional life-threatening circumstances. For example, in Ohio, when Senate Bill 127 was signed in 2017, the abortion ban prevented pregnant women from receiving abortions 20 weeks after fertilization [7]. A ban this early in pregnancy would compel pregnant women with stillborn fetuses, which the Cleveland Clinic describes as fetal death following the 20th week of gestation, to carry the nonviable pregnancy to term [8]. Following the overturn of Roe, Ohio, as well as nine other states, implemented restrictions on abortions six weeks after their last menstrual period, with nine additional states banning abortion from conception [3]. Moreover, in a group of participants surveyed in Ohio, 25% of women did not know they were pregnant until six weeks from their last menstrual period [9]. Women in this situation are denied the opportunity to elect an abortion following notice of their pregnancy, which threatens the autonomy, as well as the physical and psychological health, “of some of society’s most vulnerable” [10].

Furthermore, a multicenter, retrospective study conducted by The Consortium of Safe Labor, involving 19 U.S. hospitals, showed that patients diagnosed with fetal anomalies had slower labor progress, which led to a higher risk of emergency cesarean delivery in this cohort [11]. A slower progression of labor increases the risk of delivering a stillborn fetus due to factors such as placenta abruption, the development of infections, and other pregnancy complications such as preeclampsia [8]. Another study, which reviewed the obstetric outcomes of pregnancies diagnosed with anencephaly, a lethal condition in which cranial bones and cerebral structures are severely underdeveloped, showed that pregnant women with anencephalic fetuses are at greater risk of “redundant cesarean deliveries, polyhydramnios and associated complications, and obstetrical hemorrhage” for the birth of a nonviable fetus [12]. Additionally, it is predicted that, if all induced abortions are denied, annual pregnancy-related deaths in the U.S. will increase by 7% within the first year and by 21% in the subsequent years, with the greatest increase among non-Hispanic Black women [13]. This projected increase in pregnancy-related deaths would likely be due to a decrease in abortion providers nationwide following an increase in abortion bans [14].

Medical implications of restricting abortion care reach beyond the physical health of pregnant women. A study by Kassen et al. investigated the maternal psychological response to being diagnosed with a fetal anomaly [15]. This study demonstrated that symptoms of depression persisted toward the end of pregnancy and that “awareness of a fetal anomaly interferes with quality of life and may increase the risk of developing psychopathological symptoms” [15]. Thus, banning elective abortions for pregnant women diagnosed with fetal anomalies could lead to feelings of depression, causing lasting psychological damage to the pregnant woman.

While there is some literature available pertaining to the maternal risk of carrying a pregnancy with fetal anomalies to term, there is no current data that accurately reflects the impact of recent legislation on this patient cohort. In states where abortion is restricted, there are speculations that there will be changes in pregnancy-related death rates, decreased access to and use of prescription medications, and an increase in the physical and psychological consequences of carrying nonviable and anomalous fetuses. Hence, this study aims to investigate the medical implications of abortion bans in relation to the gestational age of the fetus, following the overturn of Roe v. Wade, on pregnant women diagnosed with fetal anomalies.

## Review

Methods

Protocol

The population/concept/context (PCC) framework was used to identify the main concepts of the review question, including abortion restrictions, fetal anomalies, and medical implications. This was then used to create inclusion and exclusion criteria for the search. Keywords were established from the PCC framework and used to generate the search strategy. Specific electronic databases were selected, and the literature was initially searched on October 2, 2022, by utilizing the developed search strategy. The Preferred Reporting Items for Systematic Reviews and Meta-Analyses extension for Scoping Reviews (PRISMA-ScR) guidelines were followed throughout the entire process [16]. Data items were extracted, summarized, and then further analyzed for result synthesis.

Information Sources

The literature was searched through three databases: Embase, Ovid Medline, and Cumulative Index to Nursing and Allied Health Literature (CINAHL) on October 2, 2022. The search strategy was developed with input from a reference and instruction librarian at Nova Southeastern University.

Eligibility Criteria

Inclusion criteria were peer-reviewed papers written in English concerning human studies published between 1980 and 2021. Date inclusion was restricted up to 2021 to include only articles prior to Roe v. Wade officially being overturned in 2022. Controlled experimental research studies that were aimed at evaluating interventions were excluded, but all others were acceptable if they met the standards of being peer-reviewed. Eligibility was featured around the PCC framework, including pregnant women diagnosed with fetal anomalies, women denied access to safe abortions, and the medical implications of this. Both maternal and fetal medical impacts were included. The types of medical implications that met inclusion criteria were maternal health effects from restricted access to safe abortions, outcomes from the lack of recommended abortions for pregnant women, and outcomes of fetal anomalies when pregnancies were carried on to birth.

To search the databases, keywords were combined in different ways by using Boolean operators of AND to combine concepts and OR to include synonyms, as well as proximity searching to ensure the results aligned with the established PCC framework strategy. Keywords used were “fetal anomal*” AND (“abortion” proximity within two words of “ban”) AND “implications”. Each of the keywords was also searched using its relevant synonyms. The keyword search was limited to only searching within the title, abstract, and author keywords to have more focused and productive results. No other limitations or filters were applied to the search. Appropriate subject headings were initially sought out by utilizing those that were assigned to relevant articles and additional ones found by searching for appropriate terms in each database’s subject heading browser.

The search was strictly done by this full search strategy, without any additional searching through reference lists of relevant articles or hand-searching journals. Final search results from each database were then exported into EndNote (n = 468), where duplicates were removed. Once completed, results were imported into Rayyan (Rayyan Systems Inc., Cambridge, MA), a systemized review collaborative database, for screening of eligibility criteria.

Selection of Sources of Evidence

After duplicates were removed (n = 136), two separate levels of screening were conducted through Rayyan. During the first screening, 11 reviewers independently screened all titles and abstracts of 332 publications to determine inclusion criteria eligibility by marking each individual article with the options of include, exclude, or maybe include. Articles determined to be excluded (n = 296) were removed, resulting in 36 full articles to be retrieved.

**Figure 1:**
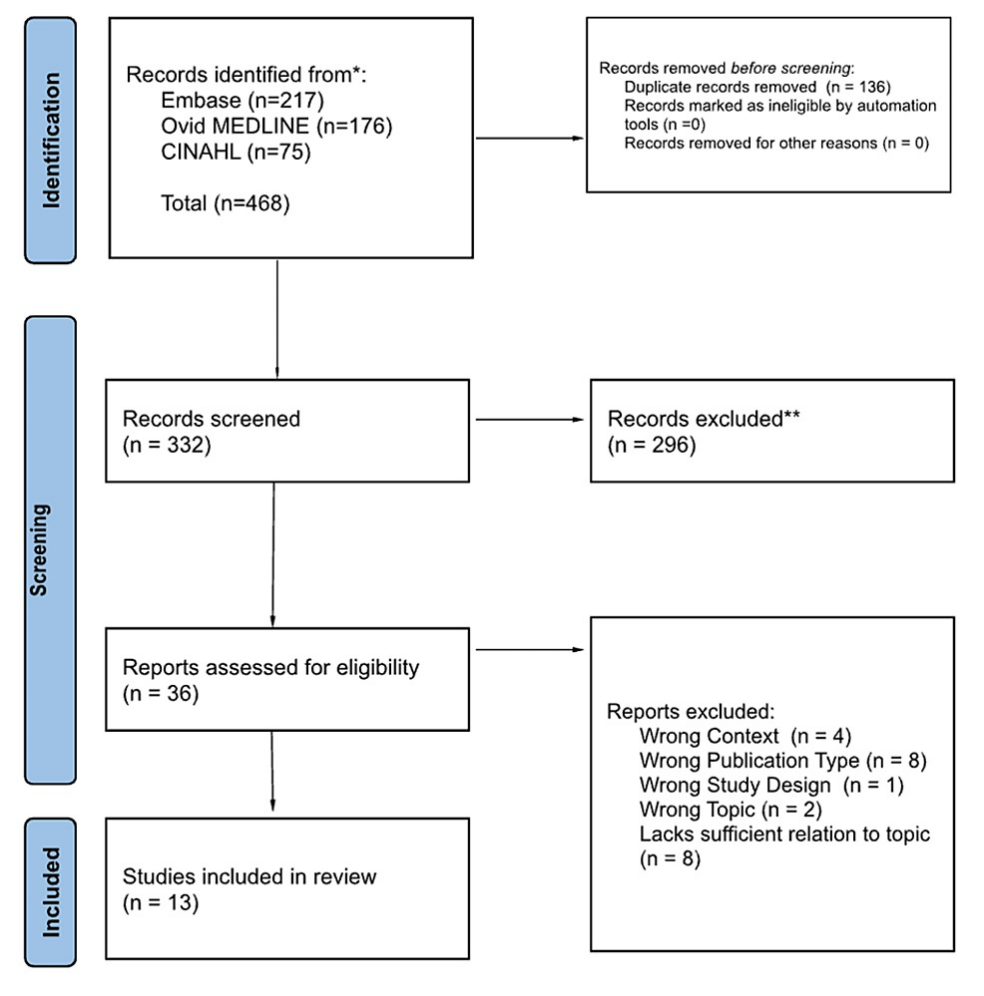
Preferred Reporting Items for Systematic Reviews and Meta-Analyses extension for Scoping Reviews (PRISMA-ScR) flowchart

The second screening process consisted of three reviewers who independently screened the full text of all articles previously determined to meet inclusion criteria and ones left undecided (n = 36). Disagreements about study selections among the three reviewers were resolved through discussion with an additional reviewer, reaching a consensus if necessary. Reasons for exclusion following the full-text screening included: wrong context (n = 4), wrong publication type (n = 7), wrong study design (n = 1), wrong topic (n = 2), and a lack of sufficient relation to the topic (n = 8). This final screening resulted in 13 articles included in the final review. Figure 1 reports the full process of study selection through the PRISMA-ScR flowchart.

Data Items and Charting

Quantitative and qualitative data from the full reviews (n = 13) were extracted and charted in Microsoft Excel independently by two reviewers. Data items were organized by the following variables: article title, study type, publication date, publication journal information (volume, issue, and page numbers), authors, abstract, country of origin, sample size, population, study purpose, and key findings. Results were discussed among the two reviewers, and any inconsistencies in the charting were resolved by a third reviewer. Data items were further utilized to synthesize results using an inductive approach through thematic analysis.

Results

Sources of Evidence

Included studies contained varying sample sizes, from one participant in a case study [17] to 2,526 participants in a retrospective cohort study [18]. These studies also provided information on fetal anomalies and abortion from a broad range of countries. Five studies (36%) originated in the U.S. [17,19-22], two (14%) from Chile [23,24], and the remaining seven studies (50%) from other countries: Sweden [18], Israel [25], Brazil [26], Poland [27], Tunisia [28], and Argentina/Uruguay [29].

Study design of the included reports displayed diversity with six (43%) retrospective cohort studies [18,20,24-26], two (14%) cross-sectional studies [27,28], two (14%) case studies [17,21] (with the latter also including a fiscal comparison), two (14%) surveys [22,29], one (7%) theoretical cohort study [19], and one (7%) semi-structured interview [23]. Additionally, there were significant fetal anomalies described within the included studies: three studies on central nervous system abnormalities [20,23,24] (with the latter two regarding anencephaly and iniencephaly, respectively), one on ectopic pregnancies [17], one on congenital diaphragmatic hernias [19], and one on cardiac anomalies [20]. Two studies, in particular, discussed multiple pregnancies [25,26] with fetal anomaly (with the latter expanding on cases of conjoined twins).

Table 1 provides details of each source included in this study.

**Table 1:** Sources of evidence characteristics

Reference (including location)	Study design	Data collection	Study aim	Findings	Recommendations	Limitations
Tucker et al. (2017), U.S. [17]	Case Study	Data were collected from patient charts with patient consent (n = 1).	To describe the case of a delayed diagnosis of a second-trimester, ectopic, abdominal pregnancy and the patient outcome	The patient’s decision to terminate the pregnancy, while overcoming perceived provider disapproval and legislative barriers, likely prevented a catastrophic intra-abdominal rupture of her misdiagnosed pregnancy.	Legislation limiting access to abortions may hurt women’s health and increase mortality.	Small sample size due to the nature of the study type.
Blomberg (1980), Sweden [18]	Retrospective Cohort Study	n = 1,263 women who were denied abortions (proband group), n = 1263 women who did not seek abortions (control group).	To investigate whether emotional stress in pregnant women might have an adverse effect in the form of malformation on fetal development.	Emotional stress in pregnant women (defined as unwanted pregnancies in this study) may interfere with fetal development and result in a higher incidence of malformation. The direct or indirect nature of this correlation is not known.	Explore ways to limit the impact of confounding variables such as the incidence of malformations increasing with lower social class, to ensure that malformations were not from food insecurity, poor prenatal care, or lifestyle aspects.	Results may not apply to the general world population since it occurred in Sweden, and cultural impacts may have influenced the results. Data used for the study was from 1960, so results may not be able to be applied to current times due to attitudes towards abortion changing in the past few decades.
Bullard et al.(2019), U.S. [19]	Theoretical Cohort Study	n = 921 women diagnosed with fetal congenital diaphragmatic hernia.	To estimate the effect of 20-week abortion bans on maternal and consequent neonatal health outcomes and costs in the setting of fetal congenital diaphragmatic hernias.	Congenital diaphragmatic hernias affect 1 in 2,200 births with mortality up to 75% (29% of these are associated with chromosomal abnormalities). Surviving children endure associated “pulmonary, neurologic, gastrointestinal, and nutritional complications.” Congenital diaphragmatic hernias are often not definitively diagnosed until 22 weeks gestation. For the cohort, the calculated cost of the abortion ban was estimated at $159,419,623 and significantly decreased happiness/quality of life for the pregnant women. The 20-week bans were associated with worse health outcomes from the maternal perspective and increased costs across all ranges.	The 20-week abortion bans are not cost-effective in the case of congenital diaphragmatic hernias. Similar economic effects on other severe anomalies would need to be explored separately.	The reliability of this model depends on assumptions made and estimates found in the literature. The model itself may oversimplify the outcomes of these pregnancies and the effects of the policies in place.
Henkel et al. (2011), U.S. [20]	Retrospective Cohort Study	n = 1,983 patients undergoing an anatomy ultrasound in 2017 at a tertiary referral center.	To quantify the likelihood of assessing all mandated fetal views during the second-trimester anatomy ultrasound prior to the proposed federal 20-week abortion ban.	For patients receiving their “anatomy ultrasound” prior to 20 weeks gestation, the risk of incomplete initial views increased and was indirectly correlated to gestational age. There were 6.4% with fetal anomalies with 38% of those diagnosed on another follow-up ultrasound. About one in five with anomalies chose to terminate the pregnancy (with cardiac and central nervous system being the most common anomalies found). Overall, moving the “anatomy ultrasound” earlier in the pregnancy would likely miss a significant number of anomalies especially impacting obese people who are less likely to have complete views before 20 weeks.	Legislation limiting abortion to 20 weeks would impact a patient's ability to make informed decisions about their pregnancy. The need for repeat ultrasounds prior to the 20-week group may account for the delay from the initial ultrasound to the termination.	The generalizability of this study may be limited beyond the geographic area in which it was conducted. A specific institution has access to services and resources that may streamline time from diagnosis to termination, suggesting the median amount of time from diagnosis to termination may be an underestimate, particularly outside of a tertiary referral network where this study was completed.
Miller et al.(2000), U.S. [21]	Multi-Case Study; Fiscal Comparison Study	n = 514 cases of second-trimester terminations from 1990-1997, 60% of cases with a prenatal abnormality diagnosis resulted in termination.	To determine what the fiscal impact of a legislative ban on elective abortions for prenatally diagnosed abnormalities would be at a specified hospital system, Hutzel Hospital in Detroit.	The estimated cost at the Detroit Medical Center (DMC), a group of facilities including Hutzel Hospital, would be $8.5 million a year. If there were a legislative ban on elective abortions for prenatally diagnosed fetal abnormalities, the additional total cost to DMC would be $74 million. This cost would be translated to about $15.30 per year per employee working in Michigan. The article also calculated a roughly $2 billion cost increase across the U.S. if elective terminations for prenatally diagnosed abnormalities were banned. This cost would have to be budgeted appropriately.	Explore what new treatment costs would be and how advances in treatment may lessen the financial strain on the hospital system for children born with prenatally diagnosed abnormalities.	Assumes the financial costs would increase if more fetuses with abnormalities were carried to term, delivered, and required medical care after delivery. This is assuming a legislative ban on second-trimester terminations for prenatally diagnosed abnormalities was enacted. Some prenatal abnormalities may be less severe at birth and therefore incur less cost over a lifetime and cohort year.
Jayaraman et al. (2021), U.S. [22]	Survey	A 22-question survey was created and sent to genetic counselors and put into 3 groups depending on the severity of abortion laws in their state. Data from n = 113 respondents were analyzed.	To analyze the impact of abortion legislation on genetic counselors and patients.	Genetic counselors reported that legislative gestational age restrictions impact their counseling and coordinating of abortion services. They also reported that regulations limit the decision-making time frame for patients with fetal abnormalities. Genetic counselors also perceive financial and emotional burdens on their patients seeking abortion services.	Larger sample.	Small sample size.
Shepard et al. (2014), Chile, [23]	Semi-structured Interview	Semi-structured interviews were conducted with n=41 women who had abortions, n=12 partners/friends, and n=8 healthcare providers in Chile. Information on hospitalizations and maternal death related to abortions was gathered from the Ministry of Health statistics.	To explore the impact of abortion bans in Chile and its effect on health care providers and pregnant women.	Chile is one of few countries that ban abortion under all circumstances. In 2008, more than 30,000 women were hospitalized due to complications of abortions in Chile. A review of 10 years of hospital data indicated that 40% of abortions are associated with fetal abnormalities. Interviewees indicated the use of safe and unsafe methods of obtaining an abortion. Misoprostol was used in some cases with access directly related to socioeconomic status. Prosecution of these women was also correlated with socioeconomic status. Three women were propositioned for sex as payment for abortive services, one woman experienced blackmail. The abortion ban prevented healthcare providers from intervening in anomalous pregnancies including anencephalic fetuses leading to declined health in pregnant women.	Abortion should be legally available at the woman’s request, at least in the first trimester, and throughout pregnancy to protect the life and health of the woman, in case of serious fetal anomalies.	Small sample size of interviews. More women who had abortions, partners/friends, and healthcare providers should be interviewed. Many of the examples detailed in this article are the accounts of a single person on the impact of the abortion ban. Additionally, the illegality of the matter brings about issues regarding accurate testimony.
Sahid et al. (2000), Chile [24]	Retrospective Cohort Study	n = 8, cases followed post-diagnosis of prenatal iniencephaly.	To explore the obstetric management of iniencephaly in a country where elective abortion is not allowed.	Iniencephaly is the rarest form of neural tube defect, with most cases being sporadic. Other defects are associated with this condition including central nervous system (CNS) and extra-CNS-associated malformations. In nations where elective abortions are legal, termination of pregnancy with this condition is common practice due to the prognosis of the condition. In nations where elective abortion is illegal, the goal is to avoid maternal trauma and obstructed labor. Inducing labor while the cephalopelvic ratio is still adequate lowers the likelihood of maternal dystocia during labor and lowers the risk of an unnecessary cesarean section.	The sample size of women who have prenatal iniencephaly cases should be expanded to further explore the effective induction of labor in lowering the risks associated with the condition.	The small sample size is due to the infrequency of the condition.
Lipitz et al. (1996), Israel [25]	Retrospective Cohort Study	n = 36 twin pregnancies in this study. Of these, n = 23 cases with structural anomaly, and n = 13 cases with chromosomal anomaly.	To evaluate the outcome of late selective fetal termination in twin pregnancies based on combined data from eight tertiary perinatal centers.	Rates of fetal abnormality are significantly higher in both monozygotic and dizygotic pregnancies. Usually, the anomaly is discordant (in one fetus only) and selective termination of this twin is appropriate. Late (> 24 weeks) selective termination in twin pregnancies is associated with a favorable perinatal outcome in the healthy twin. Parents should be informed of this possibility in countries where the law permits late pregnancy termination.	The sample size needs to be expanded to include full-term pregnancies, as all women in the study had premature deliveries.	Participants' requests to delay the selective termination procedure interfered with the results.
Nomura et al. (2011), Brazil [26]	Retrospective Cohort Study	Data from n=30 cases were reviewed from pregnancies with conjoined twins determined to have no chance of extrauterine survival or separation observed at a hospital in Brazil from 1998 to 2010.	To describe pregnancies with conjoined twins in accordance with their request for legal termination of pregnancy.	In the case of conjoined twins with no possibility of extrauterine survival, if legal authorization for abortion was obtained then 83.3% of pregnancies were delivered vaginally compared to 16.7% by c-section. Without early intervention, delivery of conjoined twins in the third trimester was C-section 100% of the time.	Pregnancy termination in the case of conjoined twins with no chance of extrauterine survival aims to minimize the maternal risk when performed before the third trimester.	Small sample size and records from a single hospital. Data collected from 1998-2010 may not resemble modern-day data.
Zareba et al. (2019), Poland [27]	Cross-Sectional Study	n=150, between 2014 and 2016, eligible women with medical reasons for terminating pregnancy took a survey as to why they decided to end pregnancy.	To profile patients terminating pregnancies and assess plans for pregnancy.	The general profile of women were those under the age of 35 who planned pregnancy - most terminated based on genetic abnormalities. Some women changed their opinion on abortion when faced with medical implications for termination, and some women were opposed to having future pregnancies, but most women still wanted children in the future.	Larger sample size, taking surveys across more clinics within Poland and other countries (i.e., U.S.) as well.	The study took place in one clinic in Warsaw, which may not be the most generalizable population to conduct a study from.
Hamdi et al. (2016), Tunisia [28]	Cross-Sectional Study	n=100, between 2013 and 2015, a descriptive study looking at patients with ultrasound discoveries of fetal malformation cases.	To evaluate Tunish Hospital diagnoses of fetal malformations and compare to past literature in hopes to get a better future prognosis.	Diagnosis of fetal abnormalities at the hospital: 17% first trimester, 54% second trimester, 29% third trimester (literature states that usually abnormalities are detected earlier). A better future prognosis would require good staff, ultrasounds, and attention to time.	Larger sample size, a more standardized population with representation from multiple hospitals/countries where women got testing done at the same place each trimester.	A mixed population of women and women who were just there for routine ultrasounds were used, only very highly trained workers with good equipment in this study (not the most generalizable), and some women had testing done in their later trimesters at a separate center.
Gadow et al., (2006) Argentina/Uruguay [29]	Survey	n = 223 patients receiving genetic counseling (both medically referred and self-referred), n = 132 patients self-referred.	To analyze the decision-making process of couples who decided to undergo prenatal genetic analysis in an area where abortion is illegal.	Of the 132 patients who referred themselves to genetic counseling, 77.6% reported that their reason for undergoing genetic analysis was to reduce their fears of having a fetus with a genetic abnormality. Of both self-referred and medically referred couples, 68.2% reported they would contemplate illegal termination of pregnancy (TOP) if diagnosed with a severe genetic abnormality. Most couples wanted to undergo genetic testing despite not having access to legal TOP. The main reported reason was to reduce fears of their fetus having an abnormality and to receive accurate information about the fetus’ condition. A majority of participants reported that they would consider illegal TOP, despite the risks, if a severe abnormality was discovered.	A larger sample size of patients who underwent genetic counseling.	Only a portion of the original population size responded to the survey due to the sensitive nature of the questions asked.

Surveying Opinions on Abortion

Public opinion on abortion tends to be mixed. Often confounding, these personal opinions are issues regarding legality and ethics (especially those concerning religion), which also vary from country to country. Some countries, such as Chile, did not legally allow abortion under any circumstances [23,24], while others allowed abortion up to a certain gestational age or only for specific indications. In the U.S., opinions on abortion tend to vary by state. In one study [22], states were divided into Guttmacher Institute categories based on accessibility to abortion under the state’s legislation: supportive, middle ground, and hostile. However, even in “supportive” states, counselors describe bureaucratic policies regarding abortion services as “onerous to the patient” causing a huge emotional impact that negatively affects well-being [22]. 

In Chile, the opinion on abortion is negative, leading to its criminalization regardless of the circumstances surrounding the pregnancy [23,24]. Often, women in these countries turn to illegal abortion methods, both safe (such as utilizing trained professionals) and unsafe (such as utilizing untrained personnel or acquiring misoprostol on the black market) [23]. Women who obtained abortions illegally, as well as their partners, friends, and health care providers, expressed their support of providing abortion as a right during interviews, stating that “the very experience of operating in secrecy generated the worst (subjective) fears and (objective) risks” [23].

Public opinion on abortion is more moderate in some countries, such as Poland, Argentina, and Uruguay [27,29]. A study completed in Argentina and Uruguay explored attitudes toward genetic testing and termination of pregnancy (TOP) [29]. Out of 223 participants receiving genetic counseling, 132 participants self-referred to genetic counselors with the majority of participants doing so to receive accurate information about their fetus’ condition and reduce fears of their fetus having an abnormality [29]. Attitudes toward TOP were positive with 68.2% of total participants reporting that they would consider illegal TOP if a severe genetic abnormality were discovered [29]. In Poland, a study was conducted among 150 women legally eligible for pregnancy termination due to medical reasons. This study found that “political views did not influence the decision [to terminate]”. Additionally, this study ascertained that opinions on abortion changed when women were put in the circumstances themselves - less than 50% of the women supported TOP on medical grounds yet despite their views they still decided to terminate [27].

Diagnosing Fetal Abnormalities

In the U.S., it is commonplace to test for anomalies even in low-risk pregnancies per the recommendations of the American College of Obstetricians and Gynecologists (ACOG). In addition to other tests, the “anatomy ultrasound” is a customary scan of the fetus typically conducted between 18 and 22 weeks [20]. A study of 100 women with chromosomally normal fetuses were diagnosed with fetal malformations significantly later than typically expected [28]. This study was conducted by “highly trained individuals using top-quality equipment” yet most of their diagnoses occurred later than expected with 17% found in the first trimester, 54% in the second trimester, and 29% in the third trimester [28].

A study in Tunisia described the advantages of early diagnosis of malformations: 1) the ability of the pregnant woman to schedule additional examinations/appointments, 2) earlier and safer termination, and 3) reduced anxiety and earlier reassurance for the pregnant woman [28]. Problems can occur, however, when diagnosing structural anomalies is attempted too early. A study found that there was a higher risk of incomplete fetal views on initial ultrasound prior to 20 weeks gestation compared to at or after 20 weeks, leading to an incomplete anatomic survey [20]. Further, incomplete views indicate repeat testing leading to later diagnoses of malformations. Of the anomalies present in 6.4% of the 1,983 women receiving their anatomy ultrasound, 38% of them were diagnosed on a follow-up ultrasound [20]. This is especially true in women with a larger body habitus or increased BMI as almost half of all incomplete studies were attributed to maternal size [20].

Another option for diagnosing fetal abnormalities is genetic counseling, which provides parents with information about the genetic status of their pregnancy [29]. A survey of 223 participants receiving genetic counseling provided their reasoning for obtaining genetic screenings: 72.2% to reduce fear of malformation in their child, 19.7% for better preparation to receive a malformed fetus, 17.5% to help the obstetrician better follow the pregnancy, and 13% for better care for the child in case of anomaly [29]. This study also showed that the most important factor determining the decision to undergo genetic testing is the desire to obtain “accurate and reliable information” about their child [29].

Fetal Health Outcomes Related to Abortion Bans 

The quality of life of the infant may also be impacted by abortion bans, especially in infants born with anomalies that are associated with a high mortality risk. Congenital diaphragmatic hernias affect one in 2,200 births and carry a 75% mortality risk. Surviving children endure health complications, including pulmonary, neurological, gastrointestinal, and nutritional deficits [19]. Congenital diaphragmatic hernias are often not diagnosed until 22 weeks gestation, which may be beyond the gestational age restriction in some states [19].

Another example of a high-risk fetal anomaly is anencephaly. Anencephaly is a rare neural tube disorder associated with high-risk intrauterine demise. For those born alive, their lifespan typically lasts from hours to days. This disorder is also often first diagnosed during a 20-week anatomy ultrasound. In countries where abortion is legal, these pregnancies are usually terminated. In cases where abortion is illegal, labor is usually induced to reduce the risk of maternal dystocia [24].

The risk of these types of fetal abnormalities is significantly higher in monozygotic and dizygotic pregnancies. Usually, the anomaly affects only one fetus, and selective termination of this twin is often performed. Late (>24 weeks) termination is associated with a favorable perinatal outcome in the healthy twin [25].

Another potential health threat to a fetus is the effect of maternal stress in an unwanted pregnancy. A Swedish study found that the stress caused by an unwanted pregnancy resulted in a higher incidence of malformations [18]. This may be attributed to women with unwanted pregnancies being more “prone to neglect proper food intake and smoke excessively”, along with a higher risk of secondary abuse of drugs and alcohol [18].

Maternal Health Outcomes Related to Abortion Bans

Abortion bans can have profound effects on maternal health. In Chile, pregnant women who elect to terminate are limited to illegal abortion services. This resulted in more than 30,000 women being hospitalized in 2008 due to complications from abortions [23]. Some women were propositioned for sex as payment for abortion services [23]. Women with higher socioeconomic status were able to obtain “safer” methods of abortion such as using misoprostol, an oral or intravaginal medication that can induce pregnancy termination. The ban in Chile also prevented healthcare providers from intervening in cases of anomalous pregnancies which had negative impacts on maternal health [23]. For example, fetuses with anencephaly can cause obstructed labor or delivery via cesarean section. In order to prevent these complications, medical interventions may be necessary such as premature induction [24]. 

In Pakistan, women who undergo illegal abortions often face health complications. These complications included hemorrhage, sepsis, and trauma. Hemorrhage was the most common complication, occurring in 43% of women who underwent an illegal abortion [26]. A study in Brazil reviewed pregnancies with conjoined twins that had no possibility of extrauterine survival. In these cases, the majority of pregnant women chose to request legal authorization for termination of pregnancy. Permission was granted to most of these patients, but it was denied to some [26]. One case from the U.S. described a patient with a misdiagnosed pregnancy who requested an abortion. After fighting several legislative barriers and perceived provider disapproval, the patient was able to undergo an abortion, thus preventing a catastrophic intra-abdominal rupture [17].

Mental Health/Quality of Life Impact of Abortion Bans

The mental health of pregnant women has been impacted by the enforcement of abortion restrictions. One study reports that, when working with pregnant women with anomalous fetuses, genetic counselors describe feeling “angry at the lack of options for them” [22]. Working in hostile states/environments produces extreme mental and emotional exhaustion for pregnant women trying to navigate the rough terrain surrounding termination [22]. The same study detailed some of the bureaucratic policies in place even in “supportive states” such as requiring an official death certificate (if the gestational age is 20 weeks or above) and funeral arrangements to be made [22]. Participants from hostile states were more likely to report emotional barriers against them from state legislation, institution policy/guidelines, legal status, mandatory waiting period, and mandatory ultrasounds prior to termination [22].

Aside from the emotional impact of abortions, there is a long-standing impact on raising a child with severe malformations or disabilities. A study from the U.S. calculated the enforcement of an abortion ban (in this case a 20-week ban) on a population of 921 pregnant women with a congenital diaphragmatic hernia [19]. It was estimated that an abortion ban would result in about 128 more live births and was directly associated with a reduction of 674 quality-adjusted life-years (QALYs) for the mothers of these children [19].

Financial Impact of Abortion Bans

Abortion bans have been shown to not only affect the physical and emotional health of pregnant women but also their financial stability. A cohort of 921 U.S. pregnant women diagnosed with a specific anomaly - congenital diaphragmatic hernias, were calculated to have an estimated total cost of $159,410,623 secondary to a legislative ban on abortion preventing them from terminating their pregnancies [19]. This financial burden was reported to significantly decrease their quality of life and happiness [19]. Another study at the Detroit Medical Center calculated an estimated cost of $8.5 million a year if a legislative abortion ban was enacted [21]. If there were a ban on abortions for prenatally diagnosed fetal anomalies, the cost would increase to $74 million a year. This financial burden would be placed on all employees in the state of Michigan, including people who were not pregnant and received no benefit from this financial responsibility [21]. If the U.S. issued a nationwide abortion ban for prenatally diagnosed abnormalities, there would be an estimated cost of $2 billion annually [21]. Additionally, a study of genetic counselors in the U.S. reported that they perceived financial stress from their patients seeking abortion services [22]. Restricting or criminalizing abortion in the U.S. could place an even greater financial and emotional burden on patients seeking termination of pregnancies diagnosed with fetal abnormalities.

Discussion

Review of Findings

Opinions on abortion were found to vary by country - often influenced by religion and law. Within the U.S., opinions differ by state, which may be due to differences in culture, prominent religions, and political predisposition in each state. With varying opinions influencing policy changes, abortions are being banned earlier than before. However, early ultrasound detections have been found to insufficiently diagnose possible anomalies [20]. With different states banning abortions at various weeks of gestation, the restriction on abortions could negatively impact pregnant women who are not diagnosed with severe fetal anomalies until later in pregnancy. This may compel women with unwanted pregnancies to carry fetuses, which often result in miscarriages, genetic defects, and newborn deaths, to full term. Previous studies have demonstrated that Roe v. Wade offered protection to women throughout pregnancy that would have counteracted these issues arising from the restrictions on abortion access [22].

Women who are forced to continue pregnancies leading to inevitable neonatal death or newborns with severe health problems could develop significant emotional stress. Experiencing emotional trauma could impact women’s mental health long term. Some states require a death certificate and funeral arrangements for pregnancies terminated after 20 weeks of gestation [22]. This is an arbitrary ritual that can cause critical emotional distress for the woman. Bringing a pregnancy to term for fetuses diagnosed with severe disabilities can cause emotional suffering in the pregnant woman, even more so in those who desired to terminate the pregnancy but were unable to [19]. Additionally, high stress with unhealthy coping mechanisms, such as using substances of abuse, could negatively impact the fetus by developing fetal alcohol syndrome, cognitive disorders, or behavioral problems [18].

In Chile, thousands of women were hospitalized due to illegal attempts at abortion [23]. While these cases are seemingly extreme, similar cases may become more prevalent in the U.S. in states that adopt strict abortion laws. In some cases, the onus may be on the woman to contest the law to receive an abortion to avoid severe medical complications that are anticipated (e.g., intra-abdominal rupture) [17]. With restrictive abortion laws currently being implemented throughout the U.S., pregnant women have an ever-increasing threat to their health.

The likelihood of significant financial burdens for women with pregnancies diagnosed with fetal anomalies has dramatically increased since the reversal of Roe v. Wade. Considering fetuses diagnosed with congenital diaphragmatic hernias in pregnant women who were unable to receive abortions had an estimated total cost of over a million dollars, net financial strain has likely been amplified in many families since the onset of abortion bans in the U.S. [19]. Future studies should measure updated financial expenditures since abortion bans have been enacted.

Limitations of the Review Process

The review process involved two separate tier reviews through the Rayyan database to guarantee that each record met the inclusion criteria. Tier I review involved eleven reviewers screening the abstracts of all records. This large number of reviewers reduced the risk of bias in the overall review process. However, limiting the screening to only the abstract, and not the full record initially, could have caused related articles not to be included in the final review, which may pose a limitation. Tier II review involved three reviewers who reviewed the full text of tier I articles to determine whether the initial articles met the inclusion criteria. Only having three reviewers, instead of all eleven, to further review the records could be displayed as an additional limitation due to possible bias, but the decision to do so was more beneficial on account of project time restrictions. A component of the tier II review was to evaluate inconclusive articles and resolve disagreements on record selection. However, the same three reviewers were utilized for this process, increasing the possibility of bias.

Limitations of Individual Studies

Many of the individual studies included have similar limitations. Small sample sizes were a common limitation [17,22-26,29]. Additionally, some testimonies may be unknowingly less accurate based on the illegality of abortion in specific countries [23]. Some studies were based on assumptions and estimations of what was found in the literature [19]. Generalizability could be a difficulty due to differences in legality and culture in the countries being studied, in comparison to the U.S. [18,20,23-25,27-29]. In the study conducted by Hamdi et al. [28], highly trained workers with good equipment may not make this study as generalizable as it could be. Additionally, women switched centers to complete testing in later trimesters, which could also reduce standardization within the study itself. Studies completed several decades ago (i.e., articles published prior to 2000) may also not be as generalizable to the implications caused by restrictions on abortion in the U.S. today [18,25]. Data collected from a single hospital or clinic may also not be generalizable [26,27]. In the study conducted by Lipitz et al., patients requesting to delay the selective termination procedure could have interfered with the results [25].

## Conclusions

Data associated with this review suggest that stringent abortion laws will likely lead to an increase in newborns with fetal anomalies, including those associated with extremely high mortality rates. This means that more women (who unwillingly carry to term) would deliver neonates that would die soon after birth, thus causing them unnecessary health-related physical and emotional trauma. Additionally, restricting access to legal abortions will likely lead to an increased number of illegal and potentially dangerous abortions, as seen in countries outside of the U.S. These often failed abortion attempts result in harm or death of the woman and/or severe disability to the fetus. With limited access to safe, affordable, and legal abortions, unwanted pregnancies will negatively impact the physical and mental health, quality of life, and financial status of these women.

Looking forward, it is imperative that more women are represented in legislative processes. This would ensure that current legislators are not only questioned on their process of decision-making, but the voices and perspectives of those who are most affected by abortion bans would be taken into account. In such a sensitive and complex topic, it is essential that women themselves are an active part of the discussion. This would ensure that legislators are fully informed and actively questioned about all aspects of the issue at hand, leading to more just and democratically aligned laws. Ultimately, such measures could shape the future of many women.
